# Short-Term Efficacy of Exclusive Enteral Nutrition in Pediatric Crohn's Disease: Practice in China

**DOI:** 10.1155/2015/428354

**Published:** 2015-05-28

**Authors:** Youyou Luo, Jindan Yu, Hong Zhao, Jingan Lou, Feibo Chen, Kerong Peng, Jie Chen

**Affiliations:** Gastroenterology Department, The Children's Hospital, School of Medicine, Zhejiang University, Hangzhou 310051, China

## Abstract

*Aims*. The objective of this study was to compare the efficacy of exclusive enteral nutrition (EEN) and corticosteroids in inducing remission in pediatric Crohn's disease (CD) and the effects of the treatment on growth improvements. *Methods*. Data was retrospectively collected for children and adolescents newly diagnosed with CD in a referral center. Patients who were followed up for more than 2 months with mild to moderate disease were included. Basic demographics, history, physical examination, the pediatric Crohn disease activity index (PCDAI), laboratory findings, endoscopic findings, and adverse effects were recorded. Remission was defined as PCDAI < 10 points. *Results*. Ten subjects received EEN and 18 patients received corticosteroids. The median follow-up in EEN group and steroid group was 9.2 weeks and 9.6 weeks, respectively. The remission rate in EEN group was significantly higher than that in steroid group (90.0% versus 50.0%, resp., *P* < 0.05). Growth improvement, which was evaluated by changes in height for age *z*-score, was more apparent in EEN group than that in steroids group (*P* < 0.05). No adverse effects were observed in EEN group. *Conclusions*. In children with mild to moderate CD, EEN is more effective than corticosteroids in improving disease severity and growth deficiency, as well as providing less side effects.

## 1. Introduction

Crohn's disease (CD) is a chronic gastrointestinal inflammation mediated by immune system. The exact pathogenesis is still unclear. The treatment strategies for CD are complex. Till now, there is no cure for CD. Most of the medications have multiple side effects. Corticosteroids are known to have certain efficacy on inducing remission therapy in pediatric CD. However, growth impairment and risk of reduced bone mineral density are the main concern of parents and gastroenterologists when the child is on systemic corticosteroids [1, 2]. Furthermore, treatment with steroids was thought to be one of the predictors for bad mucosal healing [3], which is now considered the gold standard of therapy.

Exclusive enteral nutrition (EEN) is a kind of exclusive liquid diet with either elemental or polymeric formula. The patients are not allowed to have any other diet except plain water. Studies showed multiple advantages of EEN when it was used in treating pediatric CD. Patients on EEN not only benefit by growth and bone mass improvements, but also achieve disease remission, mucosal healing, and good immune modulation [4–7]. Moreover, studies indicated that EEN had rare adverse effects when it is compared with steroids used in introduction of remission treatments [7]. However, a meta-analysis study reported the opposite findings, which suggested that corticosteroids therapy was more effective than enteral nutrition for inducing remission of CD [8].

Since the incidence of CD is lower in Asian populations compared to Western ones, most of the studies are from Western countries. Herein we compared the efficacies and adverse effects of EEN and corticosteroids induction therapies for newly diagnosed CD in one single referral center in China.

## 2. Patients and Methods

The study patients were identified using the database at The Children's Hospital, School of Medicine, Zhejiang University. This hospital is one of the major referral centers for children with inflammatory bowel disease (IBD) in Eastern China. All subjects were under the age of 18 years and were diagnosed with CD between July 2009 and December 2014 according to ESPGHAN Revised Porto Criteria including ileocolonoscopy, upper endoscopy, biopsies, clinical manifestations, and/or radiological findings [9]. Data were collected retrospectively, including basic demographics, history, physical examination, the PCDAI, physicians' global assessment of disease activity, and laboratory findings. Erythrocyte sedimentation rate (ESR) and hemoglobin and serum albumin levels were collected in laboratory results. The disease phenotypes were classified by the Paris Classification [10]. The findings of ileocolonoscopy were evaluated by endoscopists according to Crohn's disease endoscopic index of severity (CDEIS) [11]. Remission was defined as PCDAI < 10 points. PCDAI 10 to 27.5 points defined mild disease, 30 to 37.5 defined moderate disease, and 40 to 100 points defined severe disease [10]. Endoscopic complete remission was defined as a CDEIS score <3 points, while remission was defined as a score <6. A decrease in CDEIS score of >5 points meant response in endoscopic appearance [12].

Subjects included in the study were newly diagnosed mild to moderate CD and must be followed up by IBD clinic for more than 2 months. The exclusion criteria included (1) subjects with previously administration of corticosteroids, immunosuppressive drugs, or biological agents before CD diagnosis, (2) patients who had not consecutively followed EEN or corticosteroids therapy for more than 2 months, and (3) children who could not finish daily prescribed volume of formula or corticosteroids dosage for more than 3 days. Occurrence of adverse events and patients' compliance were recorded. Patients who were given corticosteroids as induction of remission therapy were determined to be in the corticosteroids group. Steroids included prednisone and hydrocortisone. The dosages and side effects were collected. The choice of EEN or corticosteroid was based on the patients' and physicians' preference.

## 3. Statistical Analysis

Statistical analysis was performed using SPSS 17.0 (SPSS Inc., USA). Data are presented as mean ± medians (interquartile range) according to distribution normality. Parametric values were compared by the use of the* t*-test method. The chi-squared test was used for categorical variables. Comparisons were made using 2-sided significance levels of *P* < 0.05.

## 4. Results

### 4.1. Study Population

The medical database of 35 consecutively enrolled patients (13 on EEN, 22 on corticosteroids) were evaluated, of whom 28 were included for analysis. Of the 7 subjects excluded, 1 switched to other medications because of increasing severity of CD, 2 stopped EEN for the lack of compliance, and 4 lost to follow-up. The median follow-up in EEN group and steroid group was 9.2 weeks and 9.6 weeks, respectively. Clinical and demographic data for corticosteroids and EEN patients are reported in Table 1. All of the 10 subjects on EEN were on polymeric formulas, including Ensure (Abbott) and Nutren Junior (Nestle). The average caloric intake in EEN group was 117.9 ± 4.2 kcal. EEN was given orally in all patients. Of the 18 patients on steroids, 15 were given prednisone; 3 were firstly treated with hydrocortisone for 14 days and then with prednisone. The average dosage for prednisone was 1.1 ± 0.4 mg/kg. The average dosage for hydrocortisone was 8.7 ± 2.3 mg/kg.

At the time of diagnosis, the two groups were not statistically different on sex, age, disease behavior, PCDAI, height for age* z*-score, and body mass index (BMI)* z*-score. Most patients in the steroids group had ileocolonic disease.

### 4.2. Clinical Evaluation

After 8 weeks, the PCDAI scores in both groups were significantly decreased when they were compared with baseline (*P* < 0.001). In EEN group, the PCDAI scores were dramatically lower than those in steroid group after 8 weeks' therapy (*P* < 0.05). There was no significant difference of the change in PCDAI between the two groups (Table 2). Ninety percent of EEN patients versus 50.0% steroids patients achieved clinical remission after 8 weeks' therapy. The remission rate in EEN group was significantly higher than that in steroids group (*P* < 0.05).

### 4.3. Nutritional Status and Growth Evaluation

After 8 weeks' treatment, change in height for age* z*-score was higher in EEN group than that in steroids group (*P* = 0.026). However, no differences were observed in BMI recovery between the two groups (Table 2). Notably, some of the observed improvements did approach statistical significance for EEN patients. At week 8, the BMI* z*-scores of the children on EEN were higher, when they were compared with scores on baseline (*P* = 0.067).

### 4.4. Mucosal Healing

In EEN group, seven subjects had their colon involved. The baseline of the mean baseline score was 6.8 (1.6–9.3). Of the 7 cases, 5 had their colon revaluated by ileocolonoscopy after 8 weeks' nutritional therapy. The mean CDEIS score [3.9 (0–12.4)] was decreased. Unfortunately, no subjects had their colon rechecked after 8 weeks' steroids' treatment.

### 4.5. Adverse Effects

No adverse effects were observed in all of the patients on EEN. However, seventy-two percent of patients suffered from side effects (13 Cushing syndrome, 3 acne, and 2 osteoporosis) in steroids group. Significant statistical difference was observed on adverse effects between the two groups (*P* < 0.001).

## 5. Discussion

Children and adolescents are special cohorts in CD patients. They have to get adequate nutrition to maintain their growth and pubertal development. However, it was reported that up to 65% CD children and adolescents had growth failure [13]. The goal of therapy for pediatric CD is to induce remission, maintain clinical remission, and promote healthy growth. Corticosteroids, a conventional medication used in the induction of remission treatment for CD, has been less recommended in recent years because of its side effects on growth and bone mineral density, which is vital factor that affects the development of children and adolescents [14]. This study revealed that patients on EEN achieved better remission rate and had less side effects when they were compared with patients on corticosteroids. Therefore, EEN can be considered as an alternative therapeutic strategy as well as corticosteroids when a patient is diagnosed with CD.

Since EEN was firstly used as inducing remission therapy for CD, it has shown multiple advantages. Studies showed that up to 89% of newly diagnosed CD children on EEN achieved clinical remission [7, 15, 16]. In a recent retrospective study including 229 patients, the investigators suggested that EEN patients had higher remission rate versus steroid patients [17]. Herein what we observed in this study revealed similar efficacy. Children on EEN can benefit from improvement of linear growth, skeletal health, and lean body mass [18]. As shown in this study, patients on EEN achieved better linear growth improvement than those on steroids. Evidences revealed that up to 85% of children and adolescents diagnosed with CD had growth failure and impaired nutritional status, and up to 40% of these patients continue to suffer from growth deficiency during the course of the disease [19, 20]. Chronic calorie insufficiency, chronic treatment with corticosteroids, and direct cytokine effects are implicit in the physiopathology of growth deficiency in pediatric IBD [21, 22]. EEN, as a therapeutic approach to CD, can not only provide sufficient calories, but also improve children's bone health [23]. Furthermore, EEN has few side effects. In this study, we found that EEN was well tolerated. All the 10 patients on EEN had no side effects. In a randomized controlled open-label study [7], abdominal pain, nausea, and diarrhea were reported as side effects in children on EEN. However, all the symptoms were slight.

Compliance is a big concern of physicians for the patients on EEN. It is reported that polymeric formulas are better tolerated and more cost effective, as well as of similar efficacy, when compared with elemental formulas [24]. So we choose oral polymeric diet as the main formula provided in our study. As a result, two subjects (2/13, 15.4%) stopped EEN because they could not take enough calories every day and refused using nasogastric tube.

There are several limitations in this study. Firstly, retrospective study derives some limitations. It would be ideal if all the patients had ileocolonoscopy to evaluate their mucosal healing after 8 weeks' therapy. And selection bias could not be avoided in this study. Another limitation in our study is the number of patients. Although the incidence of IBD in Asian countries is increasing in recent years, it is still lower than that in Western countries. In this single center study, we found that EEN can improve BMI* z*-score but could not reach statistical significance. Patients' number might be the main reason for this result. Therefore, larger multicenter perspective studies should be encouraged.

In conclusion, despite these limitations, this study provides important support for taking EEN as primary therapy in pediatric CD, including superior efficacy, less side effects, and more growth improvement when compared to steroids.

## Figures and Tables

**Table 1:** Baseline characteristics of the study population.

	Corticosteroids	EEN	*P* value
*N*	18	10	
Age at diagnosis (yr)	11.6 (1–16)	11.1 (5–15)	**NS**
Males (%)	9 (50)	7 (70)	**NS**
Disease location (%)			
Ileal (L1)	0 (0)	3 (30)	**NS**
Colonic (L2)	2 (11.1)	1 (10)	**NS**
Ileocolonic (L3)	16 (88.9)	3 (30)	**0.003**
L4a	0 (0)	1 (10)	**NS**
L4b	11 (61.1)	2 (20)	**NS**
L4a + L4b	2 (11.1)	4 (40)	**NS**
Disease behavior (%)			
B1	18 (100)	9 (90)	**NS**
B2 (stenotic)	0 (0)	1 (10)	**NS**
PCDAI	28.1 ± 7.4	23.3 ± 5.4	**NS**
Mean HFA *z*-score	−0.71	−0.53	**NS**
Mean BMI *z*-score	−2.37	−1.59	**NS**

EEN: exclusive enteral nutrition; NS: not significant; PCDAI: pediatric Crohn's disease activity index; HFA: height for age; BMI: body mass index.

**Table 2:** Comparison of PCDAI, nutritional status, and growth recovery.

	Corticosteroids (*n* = 18)	EEN (*n* = 10)	*P* value
PCDAI			
Baseline	28.1 ± 7.4	23.3 ± 5.4	**NS**
After 8 weeks	10.4 ± 11.8	2.8 ± 5.1	**0.025**
Change in PCDAI	16.4 ± 12.9	20.5 ± 6.5	**NS**
HFA *z*-score			
Baseline	−0.69 ± 1.12	−0.53 ± 1.58	**NS**
After 8 weeks	−1.06 ± 1.21	−0.43 ± 1.51	**NS**
Change in HFA *z*-score	−0.35 ± 0.53	0.10 ± 0.38	0.026
BMI *z*-score			
Baseline	−2.37 ± 1.49	−1.59 ± 1.50	**NS**
After 8 weeks	−1.18 ± 1.76	−0.35 ± 1.32	**NS**
Change in BMI *z*-score	1.18 ± 1.46	1.23 ± 0.69	**NS**

EEN: exclusive enteral nutrition; NS: not significant; PCDAI: pediatric Crohn's disease activity index; HFA: height for age; BMI: body mass index.
